# Stimulation of GABA-Induced Ca^2+^ Influx Enhances Maturation of Human Induced Pluripotent Stem Cell-Derived Neurons

**DOI:** 10.1371/journal.pone.0081031

**Published:** 2013-11-22

**Authors:** David J. Rushton, Virginia B. Mattis, Clive N. Svendsen, Nicholas D. Allen, Paul J. Kemp

**Affiliations:** 1 Divisions of Pathophysiology & Repair and Neuroscience, School of Biosciences, Cardiff University, Cardiff, United Kingdom; 2 Regenerative Medicine Institute, Cedars-Sinai Medical Center, Los Angeles, California, United States of America; Dalhousie University, Canada

## Abstract

Optimal use of patient-derived, induced pluripotent stem cells for modeling neuronal diseases is crucially dependent upon the proper physiological maturation of derived neurons. As a strategy to develop defined differentiation protocols that optimize electrophysiological function, we investigated the role of Ca^2+^ channel regulation by astrocyte conditioned medium in neuronal maturation, using whole-cell patch clamp and Ca^2+^ imaging. Standard control medium supported basic differentiation of induced pluripotent stem cell-derived neurons, as assayed by the ability to fire simple, single, induced action potentials. In contrast, treatment with astrocyte conditioned medium elicited complex and spontaneous neuronal activity, often with rhythmic and biphasic characteristics. Such augmented spontaneous activity correlated with astrocyte conditioned medium-evoked hyperpolarization and was dependent upon regulated function of L-, N- and R-type Ca^2+^ channels. The requirement for astrocyte conditioned medium could be substituted by simply supplementing control differentiation medium with high Ca^2+^ or γ-amino butyric acid (GABA). Importantly, even in the absence of GABA signalling, opening Ca^2+^ channels directly using Bay K8644 was able to hyperpolarise neurons and enhance excitability, producing fully functional neurons. These data provide mechanistic insight into how secreted astrocyte factors control differentiation and, importantly, suggest that pharmacological modulation of Ca^2+^ channel function leads to the development of a defined protocol for improved maturation of induced pluripotent stem cell-derived neurons.

## Introduction

Induced pluripotent stem cells (iPSCs) from human patients with well-defined, and often genetically determined neuronal pathology, have huge potential both for disease modelling and for reliable high-throughput drug screening. Consistent and reproducible generation of functional neurons from iPSC is crucial to the development of cellular models of neurological disease. Although a plethora of protocols are available which might produce such neuronal models, only a few have been driven by robust functional characterisation of neuronal maturation and synaptogenesis [1-3], which limits their utility for both disease modelling and the development of novel therapies *in vitro*.

Pluripotent stem cells (PSCs) differentiate into neurons through a program of neuralisation, neuronal-subtype fate specification, cell cycle exit, post-mitotic neuronal differentiation and functional maturation of regulated excitability. Despite significant advances in the development of protocols for PSC neuralisation and neural progenitor fate-specification [4], the latter stages of neural differentiation are still difficult to control. In particular, developmental studies are confounded by on-going progenitor proliferation and neurogenesis in long-term cultures, and the protracted time in culture required for differentiated neurons to become functionally mature, often exceeding 100 days [5-7]. Some successful attempts have been made to synchronise the neurogenesis of plated progenitor cells. For example, inhibition of Notch signalling, using the γ-secretase inhibitor DAPT (N-[N-(3,5-Difluorophenacetyl)-L-alanyl]-S-phenylglycine t-butyl ester), has been used to promote cell cycle exit [8]. Additionally, recent small molecule screens have identified the receptor tyrosine kinase inhibitor, SU5402, and the GSK3β inhibitor, CHIR99021, which together with DAPT effectively promoted post-mitotic nociceptor neural differentiation [9]. 

In part, the slow yet progressive nature of neuronal maturation in differentiating PSC cultures may be due to the delayed emergence and development of astrocytes [10,11]. Glia/neuron interactions have major positive effects on the functional maturation of neurons *in vitro*; both co-culture with astrocytes, and treatment with astrocyte conditioned medium (ACM), have been shown to enhance neuronal synaptogenesis [12-14]. The astrocyte-secreted factors that accelerate neurogenesis, promote neurite outgrowth and enhance the synaptogenesis of cultured neurons include thrombospondins, transforming growth factor β (TGFβ), WNTs, glial-derived neurotrophic factor (GDNF) and the chemokine C-C motif ligand 5 (CCL5) [12,15,16]. Similarly, the maturation of human PSC-derived neurons is promoted by glial co-culture or ACM [1,17], although the underlying physiological mechanisms have yet to be determined. 

Here, the effects of ACM on the electrophysiological properties of maturing human iPSC-derived neurons have been investigated. Neuronal maturation was characterized by a hyperpolarised resting membrane potential (V_m_) and the transition from a capacity to fire only evoked action potentials to spontaneous activity as a consequence of *in vitro* synaptogenesis. Fundamental to these processes in other systems is the precise regulation of Ca^2+^ homeostasis and the developmental regulation of voltage-gated ion channels. Indeed, enhanced L-type voltage-gated Ca^2+^ channel activity promotes mouse progenitor cell neurogenesis [18], and changes in L-type and N-type Ca^2+^ channel functional expression have been implicated in mouse ESC-derived neuronal differentiation [19]. Furthermore, astrocytes increase N-type channel expression in adult hippocampal cultures [20], where Ca^2+^ influx through L-type and N-type Ca^2+^ channels has been implicated in excitation-coupled neurogenesis [21]. Based on these data, it was hypothesized that one mechanism by which ACM might promote neuronal maturation is through the upregulation of voltage-gated Ca^2+^ channel activity.

A further neuromodulatory pathway which is active in immature and differentiating neurons is excitatory γ-amino butyric acid (GABA) signaling [22-24] We have previously reported that human PSC-derived neurons show ubiquitous Ca^2+^ responses to GABA, even at early stages of differentiation [7,25], an observation similar to that reported in mouse neuroepithelial cells [26]. Early GABA_A_-evoked Ca^2+^ responses, ahead of synaptogenesis, have also been observed in other systems, including retinal neurogenesis, where it was proposed that GABA might act as a trophic factor by activating L-type Ca^2+^channels [27]. Given the established excitatory role of GABA in early fetal development [24,26,28], the role of GABA in the regulation of the maturation of PSC-derived neurons was also investigated in this study.

The data presented herein suggest that functional maturation of iPSC-derived neurons is dependent upon an active GABA_A_ receptor/Ca^2+^ channel pathway. Importantly, they also strongly suggest that direct manipulation of Ca^2+^ influx and/or Ca^2+^ channel activity could provide a simple and convenient strategy to accelerate the functional maturation of immature PSC-derived neurons *in vitro*.

## Materials and Methods

### Ethics statement

Cardiff University's Biological Standards Committee performs the functions of the Animal Welfare and Ethics Body, as required by the UK's Animals (Scientific Procedures) Act 1986, in relation to its ethical oversight of the use of animals for scientific purposes. Cardiff University is authorized to carry out such work under Establishment License 30/2305, granted by the UK Home Office. The humane killing of animals for scientific purposes in the UK is authorized by Schedule 1 to the Animals (Scientific Procedures) Act 1986, which specifies humane methods according to the species, size and stage of development of the animal. All animals in this study were killed humanely in accordance with this guidance by persons registered with the University as trained and competent in these methods, and thus no specific project license authority was required. 

### Pluripotent stem cell culture and neuronal differentiation

The human iPSC line HD33i [7] was used throughout. These cells were originally generated as a control iPSC line for an unrelated study [7]. Briefly, human fibroblasts were reprogrammed by lentiviral transduction of the six transcription factors, Oct4, Sox2, Klf4, cMyc, Nanog, and Lin28 as previously described [29]. Neural stem cell lines were generated by collagenase treating (1 mg.ml^-1^, Life Technologies, Paisley, Strathclyde, U.K.) iPSC colonies, lifting them from the feeder layers and plating directly into Stemline neural stem cell expansion medium (Sigma-Aldrich, Poole, Hants., U.K.) supplemented with 100 ng.ml^-1^ FGF2 (GF003, Merck Millipore, Billerica, MA, U.S.A.), 100 ng.ml^-1^ EGF (GF144, Merck Millipore, Billerica, MA, U.S.A.), and 5 μg.ml^-1^ heparin (Sigma-Aldrich, Poole, Hants., U.K.) in polyhema-coated flasks to prevent attachment (Sigma-Aldrich, Poole, Hants., U.K.). iPS cell-derived neurospheres were expanded as spherical aggregates, termed EZ-spheres, and passaged weekly by chopping using an automated tissue chopper (McIllwain, Mickle Lab Engineering, Gromshall, UK) to ~200 µm fragments [30]. For neuronal differentiation, EZ-spheres were gently dissociated using accutase (A6964, Sigma-Aldrich, Poole, Hants., U.K.) and plated onto glass coverslips pre-coated with poly L-lysine (100 µg.ml^-1^, Sigma-Aldrich, Poole, Hants., U.K.) and laminin (50 µg.ml^-1^, Sigma-Aldrich, Poole, Hants., U.K.). Every 3 days, half of the differentiation medium (DMEM:F12 (1:3, Life Technologies, Paisley, Strathclyde) supplemented with 2% B27+ (Miltenyi Biotec Ltd., Bisley, Surrey, U.K.), 1 % non-essential amino acids (Life Technologies, Paisley, Strathclyde, U.K.), 10 ng.ml^-1^ brain derived neurotrophic factor (Peprotech, London, U.K.), 10 ng.ml^-1^ glial derived neurotrophic factor (Peprotech, London, U.K.) and 200 µM ascorbic acid (Sigma-Aldrich, Poole, Hants., U.K.) in which the differentiating iPSCs were being cultured was removed and replaced with the same volume of fresh medium. For the first 7 days 10µM DAPT (Sigma-Aldrich, Poole, Hants., U.K.) was added to the differentiation medium, after this the cells were cultured in just differentiation medium for a further 14 days. Therefore the cells underwent differentiation for a total of 3 weeks.

Differentiation medium was supplemented with ACM (1:1), additional CaCl_2,_ GABA or specific ion channel modulators included: 1.2mM CaCl_2_ to raise [Ca^2+^] from 0.6mM to 1.8mM; 300µM GABA (γ-Aminobutyric acid, Sigma-Aldrich, Poole, Hants., U.K.); 10µM bicuculline (Tocris, Bristol, Avon, U.K.); 2µM nifedipine (Tocris, Bristol, Avon, U.K.); 0.1µM conotoxin (Sigma-Aldrich, Poole, Hants., U.K.); 0.1µM agatoxin (Sigma-Aldrich, Poole, Hants., U.K.)); 0.1µM SNX482 (Tocris, Bristol, Avon, U.K.); 1µM BayK 8644 (Tocris, Bristol, Avon, U.K.).

### Astrocyte isolation, culture and medium conditioning

ACM was produced from primary mouse astrocyte cultures. Striata were dissected from newborn C57Bl/6J mice. Tissue was dissected into cold Hank’s buffered saline (HBS, Peprotech, London, U.K.), fragmented by gentle trituration, washed in HBS, and then digested by incubation with accutase (PAA Labs, Yeovil, Somerset, U.K.) and DNAse1 (0.1mg.ml^-1^, Sigma-Aldrich, Poole, Hants., U.K.) for approximately 30 minutes at 37°C. Digestion was stopped by addition of astrocyte growth medium (DMEM supplemented with 1% Glutamax (Life Technologies, Paisley, Strathclyde, U.K.), 10% fetal bovine serum (Sigma-Aldrich, Poole, Hants., U.K.) and 1% of a cocktail of penicillin, streptomycin and fungizone (Anti-Anti, Life Technologies, Paisley, Strathclyde, U.K.). A cell suspension was derived by further trituration using a P1000 pipette. Dissociated, cells were washed twice with culture medium and plated onto culture flasks. Cultures were passaged at a 1:6 ratio upon reaching confluency. Confluent cultures at P1 and P2 were used to condition neural differentiation base medium (DMEM:F12 (1:3), 2 % B27, 1% non-essential amino acids; all from Life Technologies, Paisley, Strathclyde, U.K.). ACM was harvested after 72 h, filter sterilised, aliquoted and stored for later use at -80°C. Different batches of ACM were compared using a mouse CCL2 (chemokine C-C motif ligand 2) ELISA (Quantikine Mouse CCL2/JE/MCP-1 immunoassay. R & D Systems, Abingdon, Oxon., U.K.) and normalised to a constant final concentration of CCL2 of 1 mg.ml^-1^. For differentiating neurons, ACM was mixed with differentiation base medium in a 1:1 ratio. 

### Electrophysiology

Unless otherwise stated, all reagents for electrophysiology and Ca2+ imaging were purchased from Sigma-Aldrich (Poole, Hants., U.K.). Coverslips with attached neurons were transferred to a perfusion bath mounted on the stage of an inverted microscope (Olympus CK-40, Olympus microscopes, Essex, UK) with phase contrast and rapid perfusion system capable of 20ms solution changes (Intracel RSC160, Intracel, Royston, UK). Conventional, whole cell patch clamp was performed on differentiating neurons at 1, 2 and 3 weeks post plate-down using an Axopatch 200B amplifier (Molecular Devices, Sunnyvale, California, USA) interfaced with a computer using an Axon Digidata 1320 DAC converter (Molecular Devices, Sunnyvale, California, USA). In voltage-clamp mode, voltage protocols were generated with, and evoked currents were recorded by, the Axon Instruments ClampEx acquisition software in the PClamp 9.2 suite (Molecular Devices, Sunnyvale, California, USA). Similarly, in current clamp mode, ClampEx was used to generate current protocols and to record membrane potentials (V_m_). All analyses of currents and V_m_ were performed off-line using ClampFit 9. For all recordings, pipettes were manufactured using a two-stage electrode puller (PP-830, Narishige International ltd., London, U.K.), were heat-polished using a Narishige Microforge (Narishige International ltd., London, U.K.) and had tip resistances of between 4 and 7 MΩ when filled with an the internal solution containing (in mM) 117 KCl, 10 NaCl, 11 EGTA, 2 Na.ATP and 11 HEPES. The control external solution (ESC) contained (in mM) 135 NaCl, 5 KCl, 5 HEPES, 10 glucose, 1.2 MgCl_2_ and 1.25 CaCl_2_. V_m_ and spontaneous action potentials were recorded in fast current clamp mode, where current was clamped at 0mV in ECS. Induced action potentials were recorded in fast current clamp mode during a current step protocol; current was injected to hold the membrane potential at ca. -70 mV and then 100 ms current injection was applied, starting with 0 pA and increasing to 180 pA in 10 pA increments in each successive sweep. Voltage-gated Na^+^ and K^+^ currents were measured using a voltage-step protocol; the voltage was held at -70mV and then stepped for 200 ms from -120 mV to 50 mV in 10 mV increments in each successive sweep. Leak currents were subtracted on-line using a P/N = 8 pre-pulse voltage-protocol. ESC was either supplemented with 10 mM tetraethyl ammonium chloride (TEA, a broad-spectrum K^+^ channel blocker) or NaCl was completely replaced with N-methyl-D-glutamine chloride (NMDG, a non-permeable substituent of Na^+^) to confirm dissect Na^+^ and K^+^ current from each other. The voltage-dependence of activation and steady-state inactivation of the Na^+^ currents were measured using a dual voltage-step protocol. From holding potential of -90 mV, the first 200 ms steps increased in +5mV increments to 0mV (to elicit current activation) and these were followed by a 200 ms step to 0 mV (to assess current inactivation). Following conversion to conductance (G), the G/Gmax values were plotted against the voltage to give activation and inactivation curves; the point of transection of the two curves is the voltage of peak available current, or peak Na^+^ window.

Voltage-gated Ca^2+^ currents were recorded using a voltage-ramp protocol; the voltage was held at -70mV and then ramped from -120 to 50mV over 200ms. A high BaCl_2_ (27mM) solution was used to amplify the voltage gated Ca^2+^ currents, which contained (in mM) 94.5 NaCl, 5 KCl, 5 HEPES, 10 glucose, 1.2 MgCl_2_ and 1.25 CaCl_2_. Nifedipine (2 µM) was employed to determine the contribution that L-type Ca^2+^channel channels made to the total voltage-activated Ca^2+^ currents. GABA ligand-gated (GABA_A_) currents were evoked by a 5 s application of 300 µM GABA (in ECS) at a holding potential of -70 mV or, for generating a GABA-evoked current-voltage relationship, during a voltage-step protocol similar to that used for Na^+^ and K^+^ current, as above but with each voltage step lasting for 7 s (1s before, 5 s during and 1s following the GABA application. Miniature synaptic currents were recorded during voltage clamp at -20 mV over several minutes with high analogue gain (50x). 

### Calcium imaging

Fura-2 Ca^2+^ imaging was performed using a monchromator based fluorimeter system (Cairn Research, Faversham, Kent, U.K.). Cells plated on glass coverslips were incubated in 250µl of culture medium with a final concentration of 1µl of fura-2-AM (Life Technologies, Paisley, Strathclyde, U.K.) for 30 minutes at 37°C. Coverslips were then placed in the perfusion chamber mounted on an inverted Olympus IX70 inverted microscope (Southend-on-Sea, Essex, U.K.) and continuously perfused using an RSC, rapid perfusion system (RSC160, Intracel RSC160, Intracel, Royston, UK). Fura-2 was alternately excited through a quartz, oil immersion objective with light at 340 and 380 nm (50-100ms) and re-emission from each wavelength was measured at 505 nm using a CCD camera (Hamamatsu Orca, Tokyo, Japan). 2x binning was applied to each image to increase signal intensity but at the cost of resolution. Off-line, regions of interest were circled and all intensities were background subtracted before the 340:380 emission ratio was calculated. Images were taken every 3 s.

A high K^+^ (50 mM KCl isoosmotically replacing NaCl) solution was applied for 10 s at the beginning of each experimental protocol to determine which cells expressed functional, voltage-gated Ca^2+^ channels and to establish the magnitude of the control response. Ca^2+^ channel blocking agents were applied in the same high K^+^ solution. The area under the curve for each high K^+^ application (or high K^+^ with agent) was calculated using the sum of the integrals for each data point, subtracting the integral of a line starting just before the application and ending following complete recovery.

To examine GABA_A_ responses a 10s high K^+^ was followed by a 200s rest and a 10s pulse of 300µM GABA in ECS. Any significant Ca^2+^ influx (in terms of a change in Fura-2 emission intensity ratio (i_340_/i_380_) was interpreted as an excitatory GABA response, as the cell depolarised in order to activate voltage activated Ca^2+^ channels. After 200s rest, a 10s pulse of 300µM GABA in reduced Cl^-^ solution (isosmotic replacement of NaCl with Na.isethionate in ECS, resulting in 9.9mM Cl^-^ concentration), thus cells functionally expressing GABA_A_ channels and will depolarise the cell and evoke a voltage activated Ca^2+^ influx. The background subtracted fura-2 intensity ratio traces were visually inspected for each cell, only cells showing a Ca^2+^ response to both high K^+^ and GABA with low Cl^-^ solution were included. The included cells were then sub-divided into cells with a Ca^2+^ response to GABA in ECS, considered to have an excitatory GABA response, and those without a Ca^2+^ response to GABA in ECS, considered to have an inhibitory GABA response.

### Statistical analyses

For continuous data-sets where 2 means were compared the data were checked for an approximate fit to a Gaussian distribution. Assuming an approximately Gaussian distribution a two sample unpaired, two tailed, T-test was performed. If the data distribution was significantly distinct from a Gaussian distribution then data transformations were considered in order to apply a T-test. However, if there was no obvious data transformation which resulted in an approximately Gaussian distribution then the medians were compared using a Mann-Whitney U test. For binomial data sets comparing two proportions we used two-tailed Chi^2^ tests. Statistics were reported as means or proportions followed by the sample size (number of cells) and standard error for continuous data, the probability value, test statistic and degrees of freedom (if relevant) were included for any statistical test performed. Statistics and graphical representations of the data were performed using either R (x64) 2.14.0 with the default installed libraries or Graphpad Prism version 5.01 (La Jolla, Ca. USA.).

## Results

### ACM dramatically enhances spontaneous activity of iPSC-derived neurons

**Figure 1:**
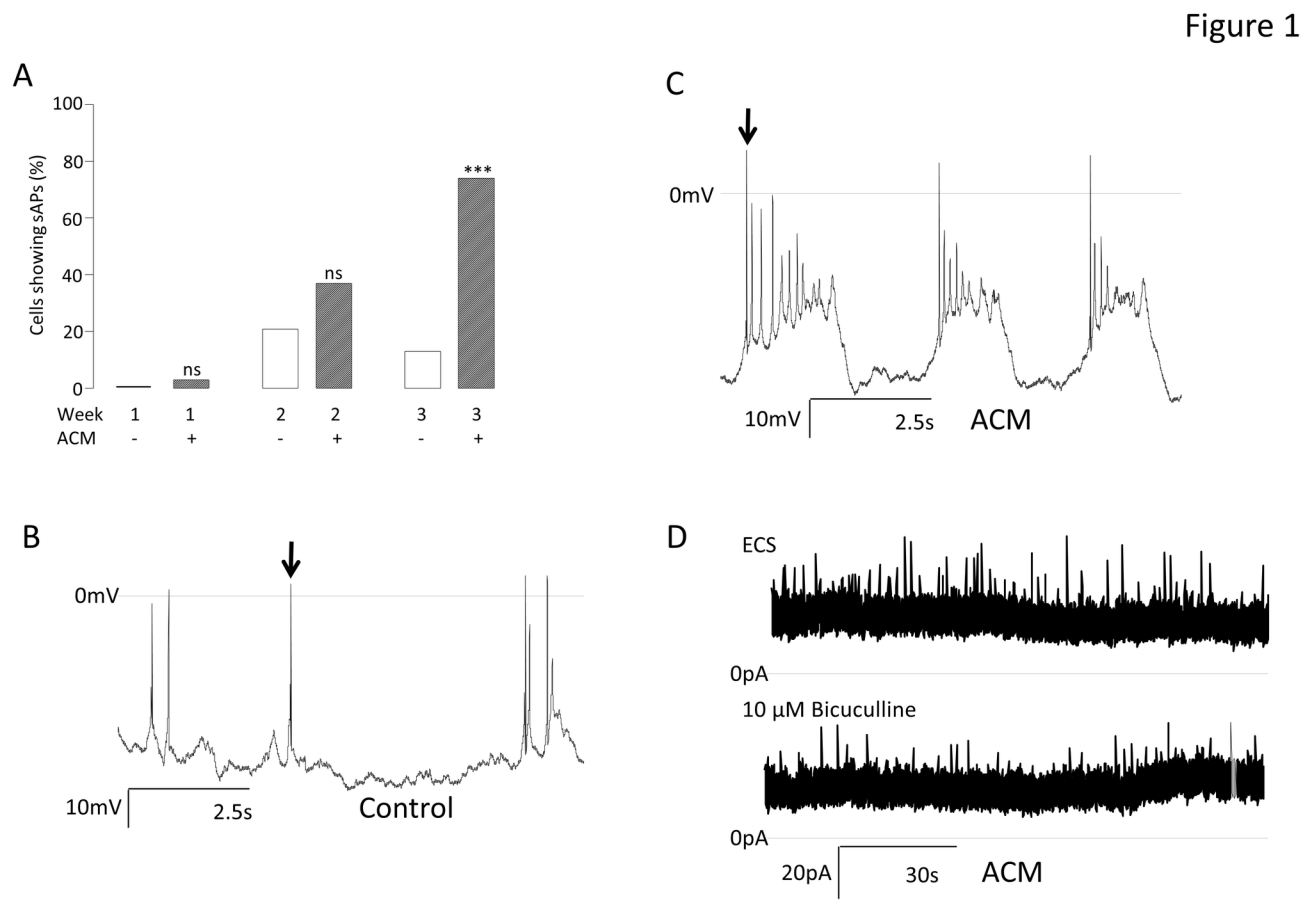
Effect of ACM on the development of spontaneous activity. A. A bar graph showing the proportion of cells (%) at each week which fired sAPs during current-clamp (I = 0 pA) at the native membrane potential (V_m_). Chi^2^ tests were performed at each week to compare control (ACM-) and ACM-treated (ACM+) iPS-derived neurons. ***P < 0.0001, ^ns^not significant; n = 147 . B. Exemplar current-clamp recording (I = 0) from a neuron differentiated for 3 weeks in control medium. Arrow indicates example action potential. C. Typical current-clamp recording (I = 0) from a neuron differentiated for 3 weeks in ACM. Arrow indicates first action potential in a spontaneous train. D. Example current recording during voltage-clamp (V_h_ = -20mV) showing tetrodotoxin-insensitive spontaneous miniature inwards currents in cells differentiated for 3 weeks in ACM in absence (upper trace) and presence (lower trace) of GABA_A_ receptor blockade with10 μM bicuculline.

In order to establish how ACM effects the electrophysiological maturity during differentiation of iPSC-derived neurons, cells were examined over the course of three weeks for spontaneous action potentials. Whether differentiated in control medium or ACM, the proportion of cells exhibiting spontaneous action potentials (sAPs) was almost zero at week 1 (Figure 1A). By week 2, this had increased significantly from 0% (n = 19) to 29 % (n = 21, P < 0.05) in the control cells and from 3 % (n = 37) to 37 % (n = 35, P < 0.0001) in ACM-treated cells (Figure 1A). The differences between control and ACM at both weeks were not significant. However, after 3 weeks, the proportion of cells demonstrating sAPs diverged dramatically and significantly (P < 0.0001), dropping to 13 % (n= 16) in control conditions but continuing to rise to 73 % (n = 19) in ACM (Figure 1A). Additionally, of the small proportion of cells in control medium which did exhibit sAPs, these were often only single events or, very occasionally, short trains of single events (see, for example, Figure 1 B). In complete contrast, a large proportion of neurons (14/19) differentiated using ACM exhibited complex spontaneous activity with trains of action potentials, often with a biphasic V_m_ and rhythmic bursting behaviour (Figure 1C). Such behaviour suggests that ACM significantly enhanced neuronal maturation and synaptogenesis. To assay synaptogenesis more directly, miniature synaptic currents were recorded in the presence of 100 nM tetrodotoxin (TTX) at either -20 mV or +20 mV. At 3 weeks, no control cells (n = 9), but 30% of ACM neurons (n = 10) exhibited inward miniature synaptic currents. 10 μM of the GABA_A_ antagonist, bicuculline, reduced the mean frequency of spontaneous inward currents from 15.5/minute to 6.5/minute, implying these were GABA-evoked synaptic currents (Figure 1D). Taken together, these data suggest a functional maturation in ACM of GABAergic neurons due to enhanced synaptogenesis.

### Of the basic electrophysiological characteristics of differentiating neurons, only V_m_ was significantly affected by ACM

**Figure 2:**
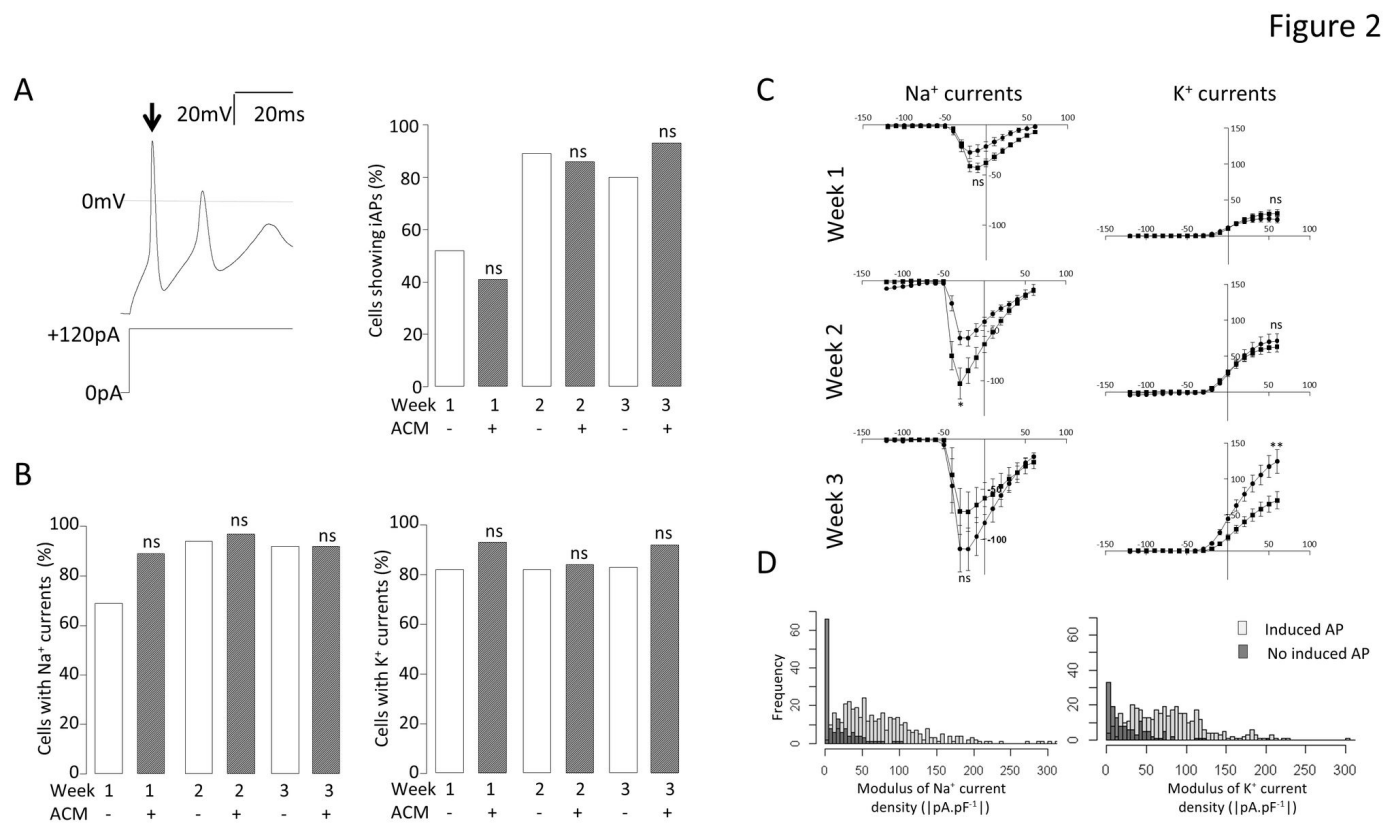
Effect of ACM on iAPs and voltage-gated Na^+^ and K^+^ currents of iPSC-derived neurons . A. Exemplar voltage recording (upper left) during a current injection of 120 pA (lower left) from a holding current which maintained V_m_ at *ca*. -70mV. Arrow indicates iAP. Right panel shows the comparison of the proportion (%) of control (ACM-) and ACM-treated (ACM+) cells which could be induced to fire an action potential by the current-step protocol at each week of differentiation. Chi^2^ tests were performed at each week to compare control medium (ACM-) and ACM-treated (ACM+) iPSC-derived neurons. ***^n^*** ^s^not significant; n = 122. B. Bar graphs comparing the proportion of cells (%) demonstrating voltage-activated Na^+^ (left) or K^+^ (right) currents in response to a voltage-step protocol. Chi^2^ tests were performed as in A, above. ***^n^*** ^s^not significant; n = 106. C. Mean Na^+^ current density (left) and K^+^ current density (right) versus voltage plots (V_h_ = -70mV) for control (circles) and ACM-treated (squares) cells during 3 weeks of differentiation. T-tests were performed to compare the mean maximal current densities of cells in control medium and ACM-treated cells at each week. *P < 0.05, **P < 0.01, ^ns^not significant; n = 106. D. Frequency histograms representing the number of cells within set ranges (5 pA.pF ^-1^) of Na^+^ (left) and K^+^ (right) current densities for all neurons. Open bars represent neurons with iAPs and filled bars represent all neurons without iAPs (n = 818).

In order to determine if this enhanced functional maturation of neurons in ACM was due to an augmentation of some of their basic electrophysiological characterisitics, cells were again examined over a three week time course, with and without ACM. Differentiation of iPSCs in either condition resulted in a rise in the proportion of cells able to fire induced action potentials (iAPs) upon current injection (Figure 2A, left) between weeks 1 and 2, from 52% (n = 21) to 89% (n = 19) in control medium and from 41% (n = 24) to 86% (n = 29) in ACM (Figure 2A, right panel). This pattern was sustained into week 3 (80% in control medium (n = 15) vs. 93% in ACM (n = 14), Figure 2A, right), where the differences between the culture conditions were not significant (P > 0.1). Thus, although ACM promoted a large increase in spontaneously active cells by week 3, this was not a direct consequence of differences in the expression of the basic cellular machinery for generation of an iAP. This idea was supported by there being no differences at any time point (P > 0.1, by chi^2^ test) in the proportion of cells expressing either voltage-dependent Na^+^ (Figure 2B, left panel) or K^+^ (Figure 2B, right panel) currents. Furthermore, although there were modest differences in maximal current densities at weeks 2 and 3 (i.e. control cells expressed smaller Na^+^ at week 2 (P < 0.05, t-test) and larger K^+^ at week 3 (P < 0.01, t-test)), these small differences did not apparently affect the ability of cells to generate iAPs (Figure 2B, C). These data are consistent with the notion that once minimum expression levels of these channels have been attained, cells are then able to generate iAPs. Indeed, upon analysing the entire cohort of cells (n = 818) using frequency histograms for cells able and unable to generate action potentials (Figure 2D), it became clear that the average functional expression of both Na^+^ and K^+^ voltage-activated channels merely had to exceed a minimal, and necessary level in order for the cell to fire an iAP. 

**Figure 3:**
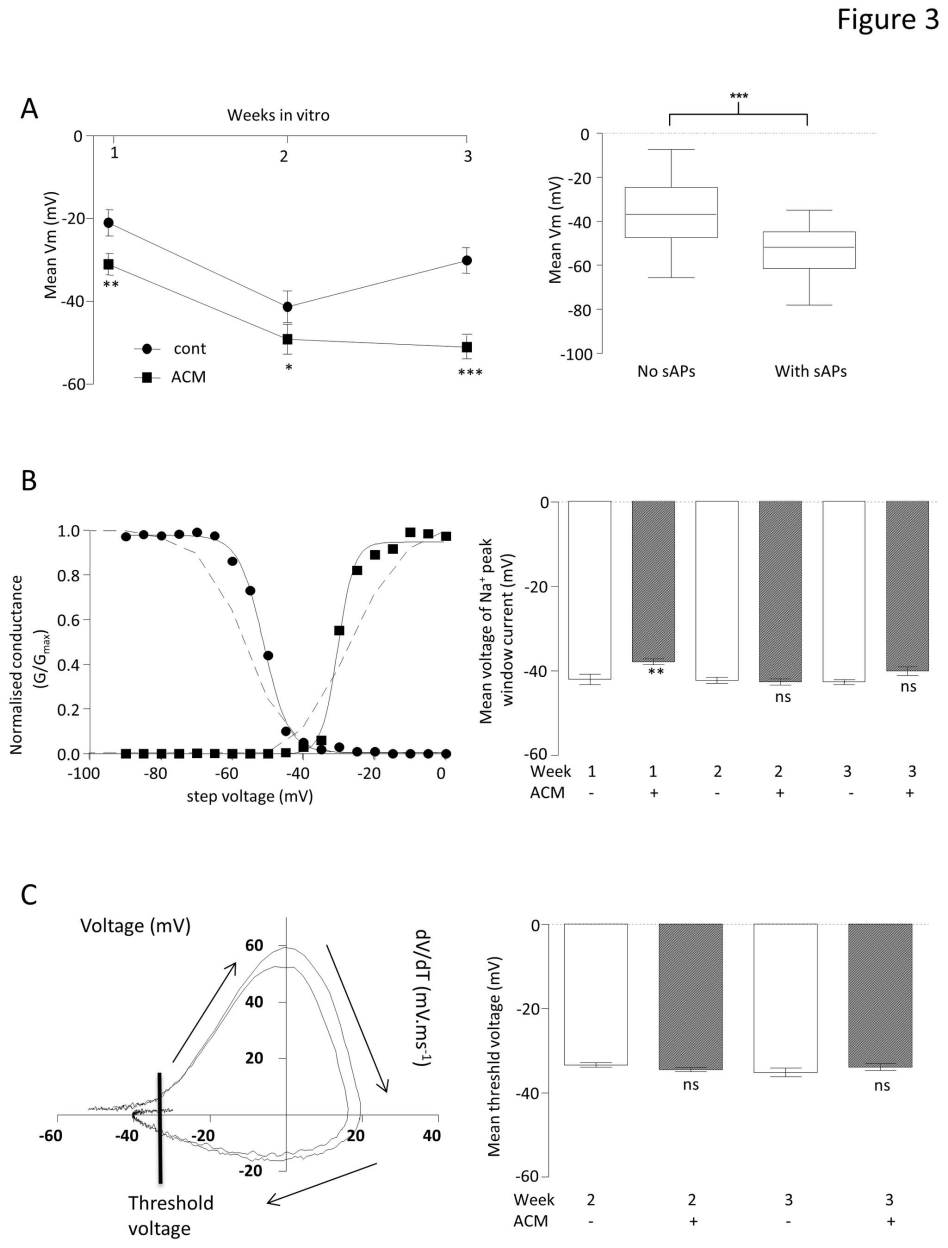
Effect of ACM on basic biophysical properties of iPSC-derived neruons . A. Comparison of mean resting membrane potentials (V_m_) of neurons differentiated in control medium (cont, circles) or ACM (squares) for up to 3 weeks. T-tests were performed at each week to compare control medium- and ACM-treated cells. *P < 0.05, **P < 0.01, ***P < 0.001,^ns^not significant; n = 147 (left). Box and whisker plots comparing all cells which either did, or did not, demonstrate spontaneous activity plotted against V_m_. Horizontal bar is the mean, box shows the 95% confidence and whiskers show upper and lower quartiles. T-test was performed to compare the two means. ***P < 0.001; n = 298 (right). B. Exemplar, normalized conductance (G/G_max_) vs. voltage plots (adjusted for series resistance and junction potential). Squares represent activation (and circles represent inactivation of Na^+^ channels. Sigmoid Boltzmann curves was fitted to both data sets (R^2^ > 0.99 for both), which intersected at the voltage at which the voltage at which the hypothetical peak Na^+^ current occured. The dashed lines are re-presentations of data from [36]showing voltage activation and inactivation curves for Na_V1.2_ (left). Bar graphs comparing mean voltages at which the peak Na^+^ window current occurs for cells differentiated in control medium and ACM for up to 3 weeks. T-tests were as in A, above. **P < 0.01,^ns^not significant; n = 77 (right). C. Rate of change in voltage (dV/dT) vs. voltage orbital plots from 2 exemplar iAPs Arrows indicate the direction of time. Line indicates voltage threshold (left). Bar graph showing the mean voltage thresholds for iAPs in ACM and control at weeks 2 and 3. T-tests were performed to compare control medium and ACM-treated cells. ***^n^*** ^s^not significant; n = 29 (right).

Other than the essential requirement for a minimal functional expression of Na^+^ and K^+^ channels, both Na^+^ current availability and resting V_m_ are crucial to spontaneous activity. At all weeks, but most strikingly at week 3, cells in ACM expressed significantly hyperpolarised V_m_ (Figure 3A left, control: week 1 = -21.0 ± 3.2 mV (n=19), week 2 = -41.3 ± 3.8 mV (n=21) and week 3 = -30.5 ± 3.1 mV (n=16), ACM: week 1 = -31 ± 2.6 mV (n=37), P<0.01; week 2 = -49.2 ± 2.2 mV (n=35), P<0.05; week 3 = -50.0 ± 2.8 mV (n=19), P<0.0001). Furthermore, across the whole data set (independent of treatment and time), those cells which generated sAPs were significantly more hyperpolarised (P < 0.0001, Figure 3A, right). Thus, ACM hyperpolarized neurons and this resulted in enhanced spontaneous activity. 

That it was the differences in V_m_ rather than inherent properties of the Na^+^ currents themselves was tested by studying Na^+^ current availability (Figure 3B). Na^+^ current activation/inactivation curves demonstrated that ACM evoked a very modest depolarisation of the peak Na^+^ current voltage (control = -42 ± 1.2 (n = 8) vs. ACM = -37.7 ± 0.7 (n = 21), P < 0.01) at week 1. However, at later time points, where the differences in spontaneous activity were most marked, ACM evoked no significant changes in peak Na^+^ current voltage (week 2: control = -42.2 ± 0.7 (n = 7) vs. ACM = -42.6 ± 0.7 (n = 21); week 3: control = -42.5 ± 0.7 (n = 10) vs. ACM = -40 ±1.1 (n = 10)). Consistent with there being few differences in peak Na^+^ current voltages, the rate of change of voltage (dV/dt) versus voltage plots showed that ACM evoked no significant changes in the threshold for iAPs (Figure 3C, control = -33.3 ± 0.56 mV (n = 6) vs. ACM = -34.5 ± 0.41 mV (n = 10) at week 2; control = -35 ± 0.98 mV (n=6) vs. ACM = -33.7 ± 0.67 mV (n=7) at week 3). Therefore, in these iPSC-derived neurons, the most important determinant of spontaneous activity is a V_m_ which is hyperpolarised sufficiently to release the Na^+^ channels from inactivation, a situation which essentially only holds true for ACM-treated cells at week 3.

Taken together, these data suggest that while ACM did not change the inherent ability of a neuron to fire an induced action potential, it increased spontaneous activity by three weeks of differentation, indicative of a more mature neuron, through an increase in the availability of Na^+^ channels because neurons were more hyperpolarized. 

### ACM enhances functional expression of voltage-gated Ca^2+^channels

**Figure 4:**
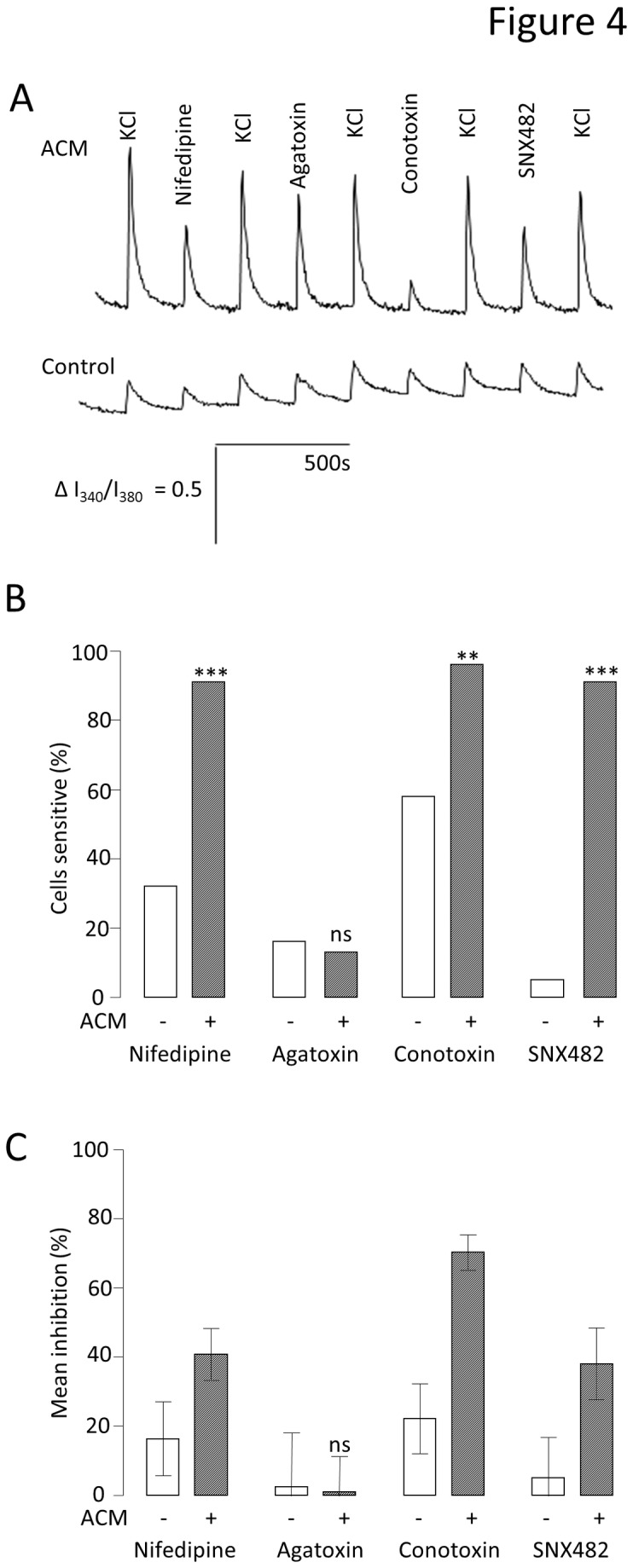
Effect of ACM on functional expression of voltage-activated Ca^2+^ channels. A. Mean traces of the ratio of intensity (I_340_/I_380_) from fura-2 Ca^2+^ imaging for control medium (solid line) and ACM-treated (dashed line) cells at week 1. Ca^2+^ influx was evoked using 50mM K^+^ solution (High K^+^) to depolarise the neurons. To measure the influence of different sub-types of voltage activated Ca^2+^channel on the depolarisation-evoked peak as a whole (the sum of the voltage activated Ca^2+^ influx) antagonists of specific channels were added to the High K^+^ solution: 10µM nifedipine (L-type Ca^2+^ channels), 100nM conotoxin (N-type Ca^2+^ channels), 100nM agatoxin (P/Q- type Ca^2+^ channels) and 100nM SNX482 (R-type Ca^2+^ channels). B. Bar graph comparing the proportion (%) of neurons which showed significant (> 5%) inhibition by each antagonist for control medium- and ACM-treated cells at week 1, compared by chi^2^ tests. ***P < 0.001, **P < 0.01, ^ns^not significant; n = 42. C. Bar graph showing the reduction of Ca^2+^ influx (mean % inhibition) elicited by each antagonist for control medium- and ACM-treated cells at week 1, compared by t-tests. ***P < 0.001, **P < 0.01, ^ns^not significant; n = 42.

Neuronal voltage-gated Ca^2+^ channels, particularly N- and L-type, are associated with the pre- and post-synaptic membranes respectively [31]. Ca^2+^ channel expression has been associated with neuronal maturation [18]. Therefore, in order to investigate further the mechanism by which ACM increases functional maturity, Ca^2+^ channel functional expression was examined over the course of the three week differentiation. Peak inward Ca^2+^ current densities (carried by Ba^2+^) at each time point were larger in the cells treated with ACM (control week 1 = 0 ± 0 pA.pF^-1^ (n = 6) vs. ACM week1 = -3.2 ± 0.6 pA.pF^-1^ (n = 15, P < 0.001); control week 2 = -1.9 ± 0.4pA.pF^-1^ (n = 5) vs. ACM week 2 = -8.7 ± 1.9 pA.pF^-1^ (n = 12, P < 0.05); control week 3 = -5.6 ± 1.3 pA.pF^-1^ (n = 8) vs ACM week 3 = -9.2 ± 1.0 pA.pF^- 1^ (n = 9, P < 0.05). To dissect the channel subtypes responsible for carrying the Ca^2+^ current at the early time points, fura-2 based Ca^2+^ imaging was employed. 50mM KCl (high K^+^) solution was used to activate all voltage-gated Ca^2+^ channels in the absence and presence of 10µM Nifedipine (L-type blockade), 100nM conotoxin (N-type blockade), 100nM agatoxin (P/Q-type blockade) or 100nM SNX482 (R-type blockade) (Figure 4A). Consistent with the whole-cell electrophysiology, cells differentiated in ACM not only produced larger responses, reflected in larger areas under the curve (AUC) for each high K^+^ application (Figure 4A), but significantly more cells responded to this stimulus. At week 1, in control medium, 89% of cells responded to high K^+^ with a mean AUC of 2.8 ± 0.4 (n = 19), whereas 100 % of cells grown in ACM responded with a mean AUC of 6.1 ± 0.7, (n = 23, P < 0.01). Furthermore, there were significant differences in the proportion of cells in each culture condition which were sensitive to nifedipine (31.3 % in control medium (n = 19) vs. 91.3 % in ACM (n = 23, P < 0.0001), conotoxin (57.9 % in control medium versus 95.7 % in ACM) and SNX482 (5.3 % in control versus 91.3 % in ACM, Figure 4B). Agatoxin sensitivity was very small, and not significantly different under the two conditions, implying that few cells expressed P/Q- type Ca^2+^ channels (Figure 4B). These data show that ACM promoted increased expression of L- N- and R-type Ca^2+^ channels. Additionally, ACM also increased significantly the magnitude of the inhibition caused by each of the specific channel blockers (Figure 4C). Thus, nifedipine-sensitive AUC went from 16.4 ± 10.7 % (n = 19) of the high K^+^ response to 40.8 % ± 7.5 % (n = 23, P < 0.001), conotoxin-sensitive AUC went from 22.2 ± 10.1 % to 70.2 ± 5.1 (P < 0.001) and SNX482-sensitive AUC went from 5.2 ± 11.7 % to 38 ± 10.4 % (P < 0.001). In conclusion, ACM not only promoted a greater proportion of the cells to express Ca^2+^ channels, but those channels also made an increased functional contribution to each neuron, again indicative of a more mature neuronal phenotype.

### ACM does not alter functional expression of ionotropic GABA currents

**Figure 5:**
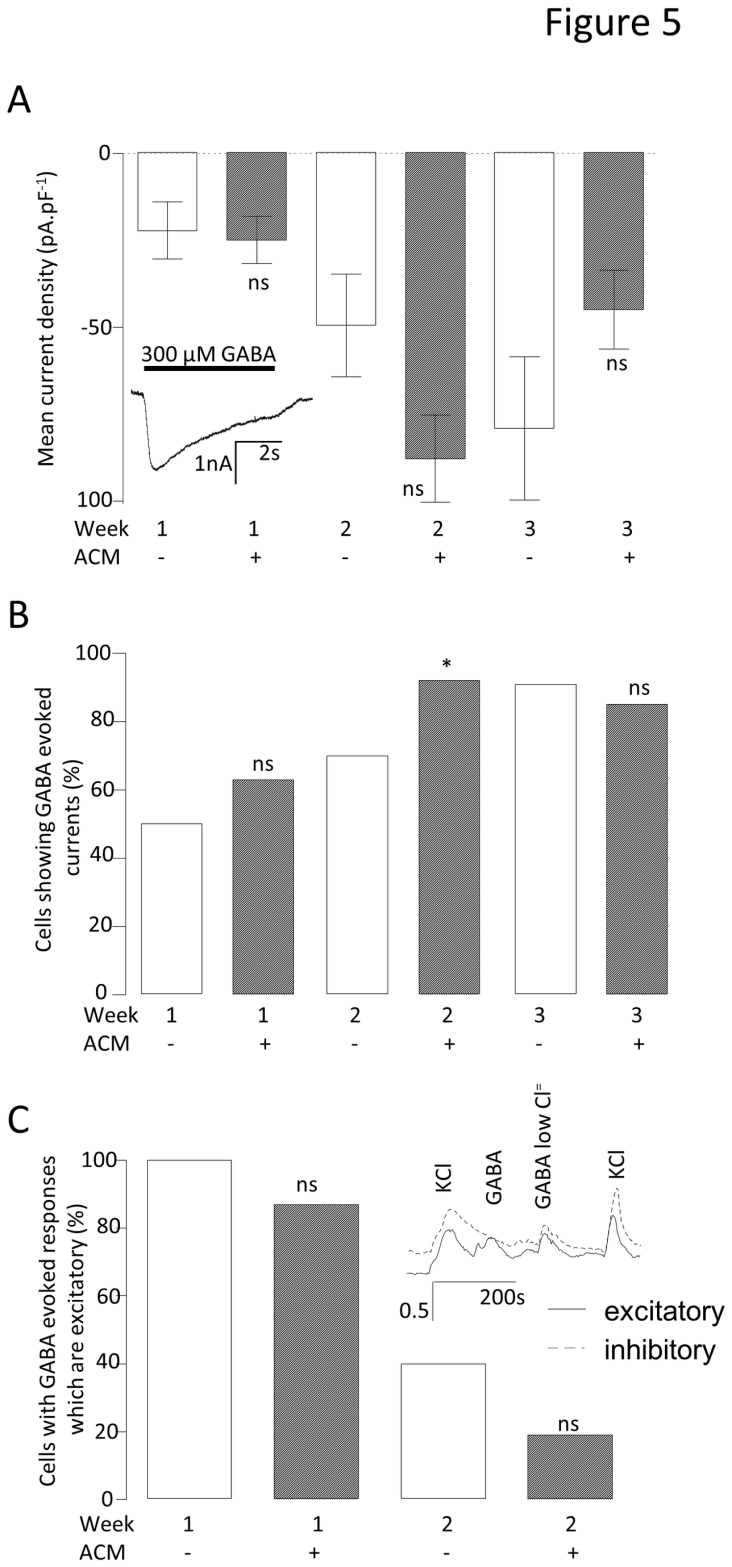
Effect of ACM on GABA_A_ function. A. Bar graph comparing the mean GABA-evoked current densities (V_h_ = -70mV) for control medium- and ACM-treated neurons. Mann Whitney U tests were performed comparing the medians (due to the data showing an extremely non-normal distribution) at each week. ***^n^*** ^s^not significant. n =59. B. Bar graph comparing the proportion of cells demonstrating a GABA-evoked current. Chi^2^ tests were performed to compare the proportion of cells with GABA currents at each week. *P < 0.01, ^ns^not significant. n =95. C. Bar graph comparing the proportion of cells with GABA-evoked current that is excitatory. Inset shows fura-2 recordings exemplifying neurons with either excitatory (solid line) or inhibitory (dotted line) GABA responses. Statistics as in B, above. *P < 0.01, ^ns^not significant. n =58.

GABA can be either excitatory or inhibitory, depending upon the neuronal Cl^-^ equilibrium potential, a parameter which is itself developmentally controlled via differential expression of several Cl^-^ transporters [24]. As these excitatory or inhibitory GABA-responsive phenotypes are indicative of neuronal immaturity or maturity, respectively, they were examined in order to establish whether ACM was increasing neuronal maturity regulation of the GABA-response *per se*. The magnitude of 300 μM GABA-evoked inward currents increased dramatically from week 1 to 2 in ACM (week 1 = -24.8 ± 6.8 pA.pF^-1^ (n = 8) to week 2 = -87.8 ± 12.5 pA.pF^-1^ (n = 20)) with smaller increases observed in control medium (week 1 = -22.1 ± 8.2 pA.pF^-1^ (n = 6) to week 2 = -49.4 ± 14.9 pA.pF^-1^ (n = 7)). From weeks 2 to 3 no significant increases were observed in the median GABA current densities in either culture condition (control = -79.1 ± 20.7 (n = 11) vs. ACM = -44.9 ± 11.4 (n = 7); Figure 5A). However, ACM did not evoke a significant difference in the magnitude of GABA-evoked currents at any week, comparing either the means or medians. Although there was a modestly higher proportion of cells which expressed GABA-evoked currents in the ACM than in control medium at 2 weeks (control = 70 % (n = 10), ACM = 92 % (n = 25), P < 0.05), the control cultures had caught up by week 3 (control = 91 % (n = 12) vs. ACM = 85 % (n = 13), Figure 5B). Using conventional patch-clamp, it is not possible to ascertain whether the cellular responses to GABA_A_ are normally excitatory or inhibitory. Therefore, Ca^2+^ influx in response to GABA was measured by fura-2-based Ca^2+^ imaging. Where the GABA response was excitatory, Ca^2+^ influx could be observed as a consequence of GABA-evoked depolarization to cause activation of voltage gated Ca^2+^ channels. If no response to GABA was observed, this could be the result of either GABA acting as an inhibitory neurotransmitter (to produce a hyperpolarization), or lack of expression of GABA_A_ receptors and/or Ca^2+^ channels. To distinguish between these possibilities, GABA was also applied in a low Cl^-^ extracellular solution; in this 9.9 mM Cl^-^ solution, GABA_A_ receptor activation can only be depolarising, and must cause Ca^2+^ influx if Ca^2+^ channels are expressed. Ca^2+^ channel expression was confirmed by application of high K^+^ to evoke Ca^2+^ influx directly by depolarisation. Using these approaches, cells that switched from an excitatory (immature) to inhibitory (mature) phenotype between week 1 and 2 were identified, but ACM did not significantly affect this switch (Figure 5C, week 1: control = 100% (n = 17), ACM = 86.7 % (n = 15) vs. week 2: control = 40% (n = 10), ACM = 18.8% (n = 16); Figure 5C). While ACM has been demonstrated to increase functional maturity of GABAergic neurons via increased Na^+^ channel function and Ca^2+^ channel expression, it does not aid in this maturity via altering the balance between neuronal excitatory and inhibitory responses to GABA.

### Neuronal phenotype is remodeled by manipulating Ca^2+^ handling

**Figure 6:**
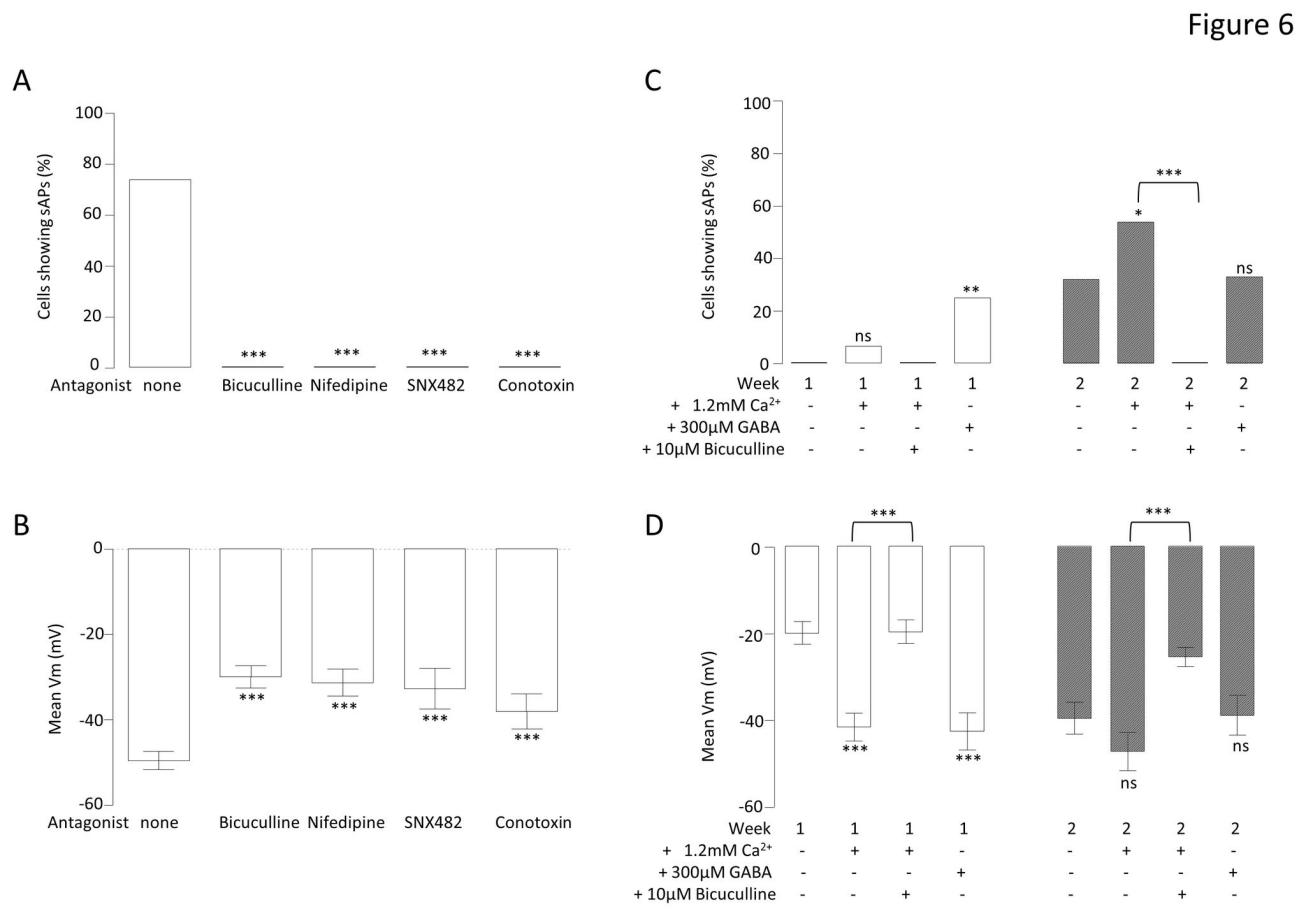
The effects of manipulating Ca^2+^ and GABA signaling in the culture medium. Bar graphs comparing the proportion of cells firing sAPs (A) and mean V_m_ (B) following 3 weeks of differentiation in ACM alone, or ACM with 10µM bicuculline (a GABA_A_ antagonist), with 2 µM nifedipine (an L-type Ca^2+^ channel antagonist), with 100 nM conotoxin (a N-type Ca^2+^ channel toxin) or with 100 nM SNX482 (an R- type Ca^2+^ channel toxin). Chi^2^ tests were performed comparing the ACM with each toxin against ACM alone. ***^±^***P < 0.05, ***P < 0.001, ^ns^not significant, n = 57. Bar graphs comparing the proportion of cells firing sAPs (C) and mean V_m_ (D) following 1 and 2 weeks of differentiation in control medium (0.6 mM Ca^2+^), high Ca^2+^ medium (1.8mM Ca^2+^), control medium with 300µM GABA and, high Ca^2+^ medium with 10µM bicuculline). T-tests were performed comparing the GABA and high Ca^2+^ media with control medium and the bicuculline medium with high Ca^2+^ medium for each week. ***^±^***P < 0.05, ***P < 0.0001, ^ns^not significant, n = 133.

The early up-regulation of voltage-gated Ca^2+^ channels by ACM would be predicted to result in long-lived and robust influx of Ca^2+^ upon stimulation. Such stimulation might well be via GABA, either at new synapses or extrasynaptically, which would then elicit an excitatory response in most cells at week 1. To investigate whether ACM might be accelerating the rate of neuronal maturation via augmentation of GABA_A_-dependent regulation of Ca^2+^ channels, cells were differentiated in ACM for 3 weeks in the absence and presence of bicuculline (GABA_A_ receptor block) or specific Ca^2+^ channel blockers. Remarkably, blockade of GABA_A_ receptors, L-type, R-type or N-type Ca^2+^ channels all significantly depolarised the neurons (from -50.8 ± 2.8 mV (n = 19) in the ACM to -27.1 ± 1.2 mV (n = 10) with 10 µM bicuculline, -21.0 ± 2.5mV (n = 9) with 2 µM nifedipine (L-type Ca^2+^ channel block), -27.3 ± 3.9 mV (n = 9) with 100 nM conotoxin (N-type Ca^2+^ channel block), and -25.5 ± 2.4 mV (n = 10) with 100 nM SNX482 (R-type Ca^2+^ channel block), Figure 6A) and abolished the ACM-evoked increases in spontaneous activity (P < 0.0001, Figure 6B). To test the idea that Ca^2+^ influx was an important determinant of neuronal maturation, extracellular Ca^2+^ concentration was increased in the control medium from 0.6 mM to 1.8 mM and both resting V_m_ and spontaneous activity measured using whole cell patch-clamp. The increased Ca^2+^ concentration resulted in an enhancement in the proportion of cells generating spontaneous activity, which became significant at week 2, from 29 % (n = 21) in 0.6 mM Ca^2+^ to 56 % (n = 16) in 1.8 mM Ca^2+^ (P < 0.05, Figure 6C). Increased Ca^2+^ also evoked significant hyperpolarisation of mean resting V_m_ at week 1 (from -21 ± 3.2 mV (n=25) in 0.6mM Ca^2+^ to -42 ± 4.5 mV (n=16), P<0.0001, Figure 6D.). By week 2, the resting V_m_ of both groups had stabilized at this hyperpolarised level (Figure 6D). These data suggested that increasing Ca^2+^ influx could partially mimic ACM. However, without a significant depolarizing stimulus, neither increased Ca^2+^ channel expression nor an increased driving force, alone or in combination, would necessarily result in increased intracellular Ca^2+^. Importantly, the ability of high extracellular Ca^2+^ to hyperpolarize the cell and augment spontaneous activity was completely ablated when GABA_A_ receptors were blocked by 10 µM bicuculline (Figure 6C, D). Addition of bicuculline to the high extracellular Ca^2+^ medium abolished spontaneous activity (from 56% (n = 16), to zero (n = 19), P<0.0001) and significantly depolarised V_m_ (from -41.4 ± 3.2 mV (n = 16) to -19.5 ± 2.7 mV (n = 19), P<0.0001). Moreover, addition of 300 µM GABA was as effective as the increased extracellular Ca^2+^ at week 1 in augmenting spontaneous activity (from 0% (n = 25) in control to 25 % (n = 8), P < 0.01, Figure 6C) and hyperpolarising the V_m_ (from -19.8 ± 2.9 mV (n = 25) to -42 ± 3.3 mV (n = 8), P < 0.001, T = 4.3, df = 31, Figure 6D). These data show that GABA-dependent depolarisation or high extracellular Ca^2+^ are both capable of mimicking the ACM-evoked maturation of differentiating neurons. However, beyond week 2, the differential effects of high Ca^2+^ or GABA are lost and both the V_m_ and rates of spontaneous activity return to levels closer to those observed in control medium. This might have been attributed to a loss of excitatory GABA stimuli. Indeed, only half of the cells in the high Ca^2+^ cultures showed an excitatory GABA response (control = 100 % (n = 17) vs. high Ca^2+^ = 53 % (n = 17), P < 0.001).

**Figure 7:**
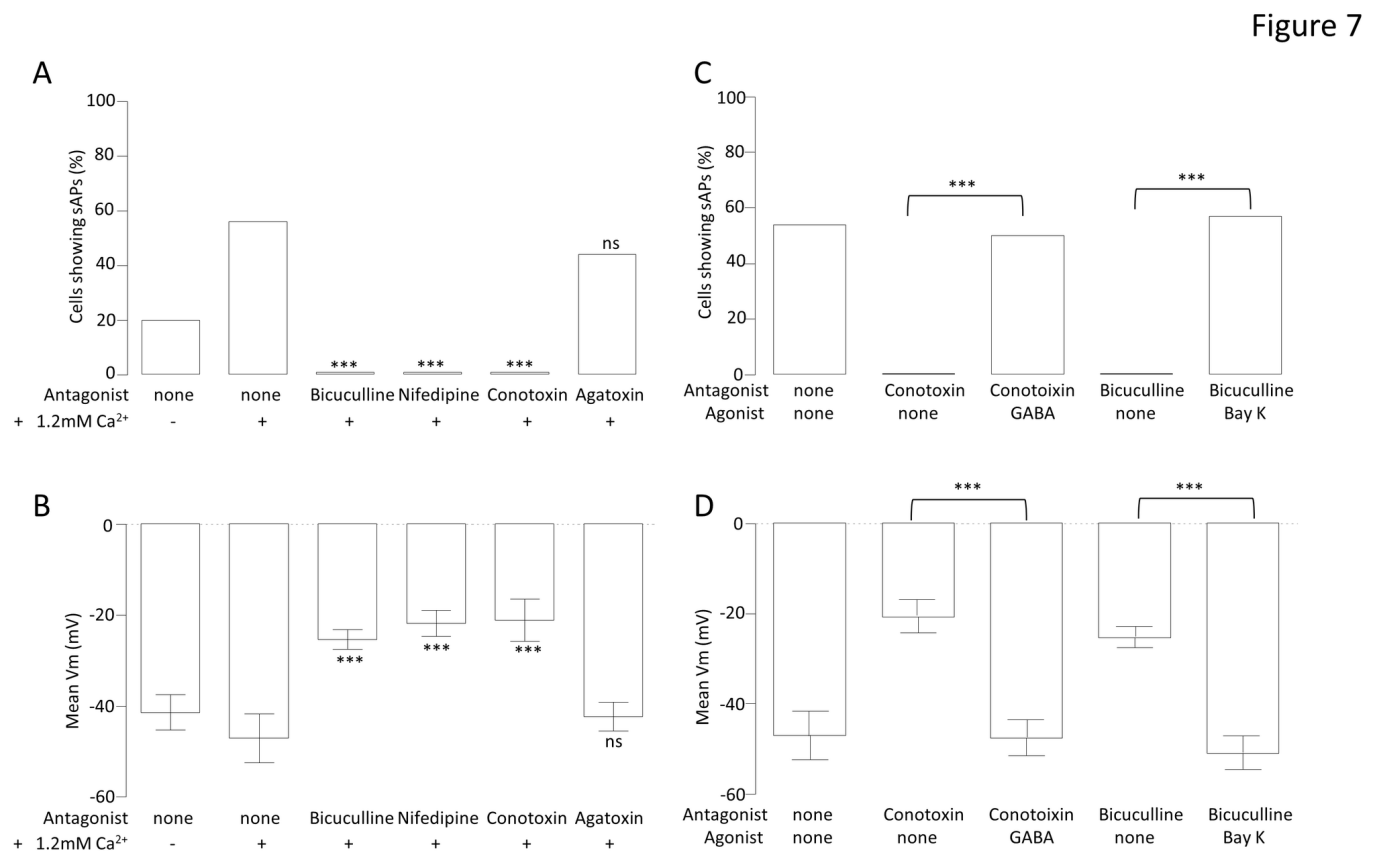
Manipulation of GABA_A_ and Ca^2+^ currents in high Ca^2+^ medium: effects and functional rescue. Bar graph comparing the proportion of cells firing sAPs (A) and mean V_m_ (B) at week 2 when treated with control medium (0.6mM Ca^2+^), high Ca^2+^ medium (1.8mM Ca^2+^) and high Ca^2+^ medium with 10µM bicuculline, with 2µM nifedipine and with 100nM conotoxin and, with 100nM agatoxin (a potent P/Q- type Ca^2+^ channel toxin). Chi^2^ tests were performed to compare the high Ca^2+^ medium with each toxin against high Ca^2+^ medium alone. ±P < 0.05, ***P < 0.0001, ^ns^not significant, n = 90. Bar graph comparing the proportion of cells firing sAPs (C) and mean V_m_ (D) at week 2 treated with high Ca^2+^ medium (1.8mM Ca^2+^) and high Ca^2+^ medium with 100nM conotoxin, conotoxin with 300µM GABA, 10µM bicuculline and, bicuculline with 1µM BayK 8644 (an agonist of L-type Ca^2+^ channels). Chi^2^ tests were performed to compare the proportion of cells with treated with conotoxin vs. conotoxin with GABA and biciculline vs. bicuculline with BayK 8644. ***P < 0.0001, n = 65.

**Figure 8:**
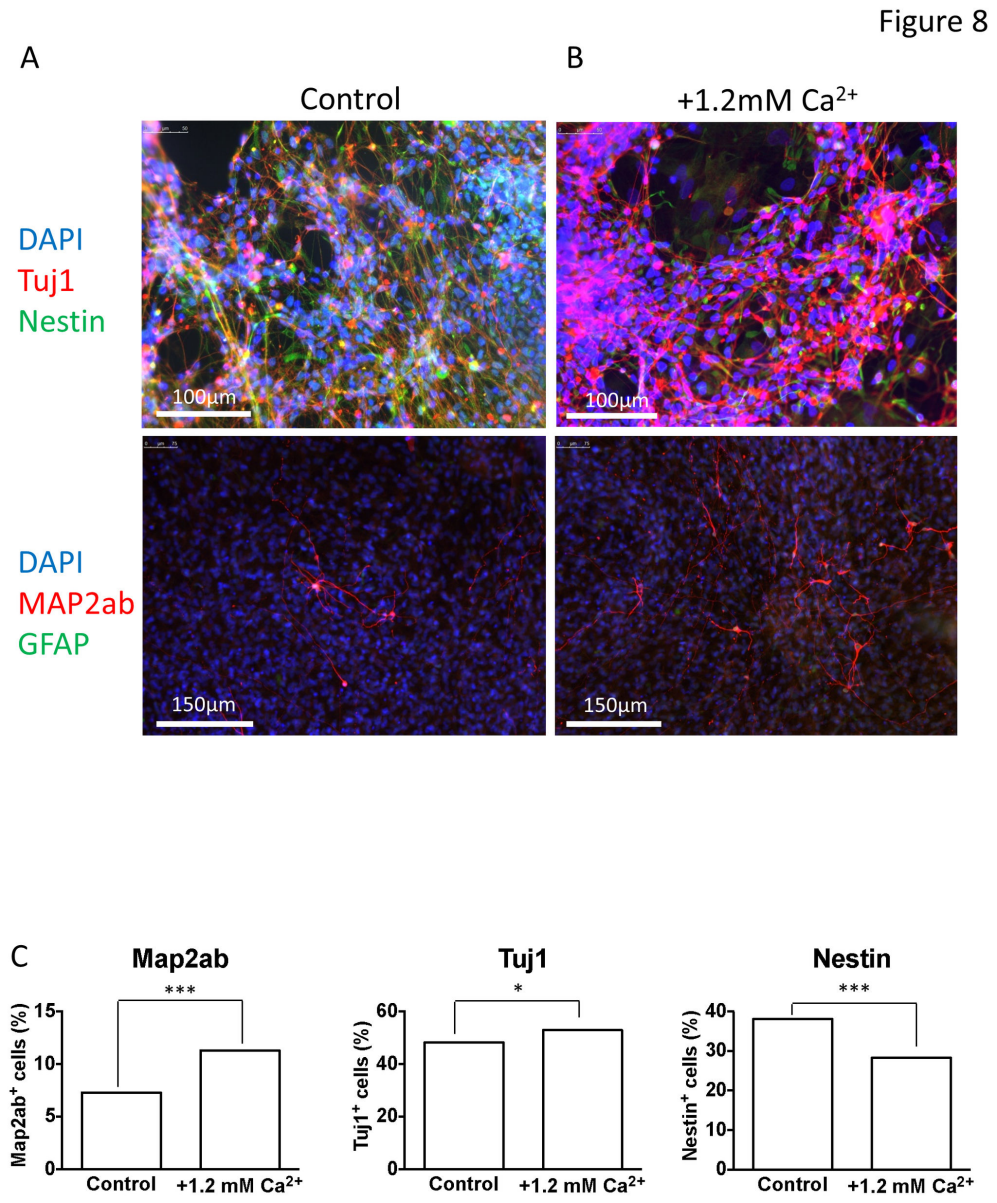
Immunofluorescent staining of cells differentiated in control and high Ca^2+^ media. Immunofluorescent staining of iPSC-derived neurons differentiated for 3 weeks in either control (A, 0.6mM) or high (B, 1.8mM) Ca^2+^ media; note that 1.8 mM Ca^2+^ was achieved by adding an extra 1.2 mM CaCl_2_ to the control medium. DAPI nuclear stain was used to show the number of cells in each field of view, in addition to primary antibodies raised against Tuj1 (neuron-specific class III β-tubulin, red in the top 2 panels), nestin (top 2 panels), GFAP (glial fibrillary acidic protein, green in the lower 2 panels) and MAP2ab (microtubule associated protein 2-ab, red lower 2 panels). C, bar graphs showing percentage Map2ab-positive (left), Tuj1-positive (middle) and Nestin-positive (right) cells, each from 4 regions of interest over 2 coverslips (approximately 2900 cells for each analysis) for both control and high Ca^2+^ differentiations at week 3, the two populations in each condition were compared by Chi squared test. ***P<0.0001, Chi^2^ = 28.04 (Map2ab) and 24.59 (Nestin); *P<4.97, Chi^2^ = 4.97 (Tuj1).

Pertinent channel blockers were employed to test whether high Ca^2+^ might augment the maturation of neurons via GABA_A_-dependent Ca^2+^ influx through specific Ca^2+^ channels. In this separate set of experiments, 2 weeks of differentiation in 1.8 mM Ca^2+^ resulted in the proportion of cells exhibiting spontaneous activity being increased from 19 % (n = 21) to 56 % (n = 16) (P < 0.05, Figure 7A) and hyperpolarizing the V_m_ (Figure 7B). Importantly, in the presence of 1.8 mM Ca^2+^, bicuculline, nifedipine or conotoxin completely blocked spontaneous activity (P < 0.001) and depolarised the V_m_ (high Ca^2+^ = -47.1 ± 5.4 mV (n = 16); bicuculline = -30 ± 2.6 mV (n = 19), P < 0.0001; conotoxin = -21.1 ± 4.6 mV (n = 15), P < 0.0001, and; nifedipine = -21.8 ± 2.8 mV (n = 13), P < 0.0001). In contrast, agatoxin was completely without affect (Figure 7A, B), suggesting that the effect of high Ca^2+^ was dependant upon L- and N- type Ca^2+^ channels and GABA signaling. In further support of the idea that high extracellular Ca^2+^ promotes neuronal maturation are the data presented in Figure 8 which show that, compared to control, iPSCs differentiated in high Ca^2+^ for three weeks demonstrate smaller nestin-positive and larger Map2ab- & Tuj1-positive populations of cells, indicative of enhanced neuronal differentiation (Figure 8).

Since either GABA_A_ receptor or N-type Ca^2+^ channel blockade diminished the positive effects of high Ca^2+^ on functional maturation, attempts were made to bypass the effects of each by activating alternative Ca^2+^ influx mechanisms. Thus, conotoxin co-treatment with high Ca^2+^ completely blocked the ability of high Ca^2+^ to augment spontaneous activity (Figure 6C) and depolarised the cells (Figure 7D). Remarkably, the effect of conotoxin was reversed by GABA (Figure 7C, D), which re-established the ability of cells to fire action potentials (from none (n = 15) with conotoxin to 50% (n = 10) with both GABA and conotoxin, P < 0.01, chi^2^ = 9.38, Figure 7C) and evoked a hyperpolarisation of the membrane (from -20.7 ± 3.5mV (n = 15) with conotoxin to -47.6 ± 3.9 mV (n = 10) with both GABA and conotoxin, P < 0.0001, Figure 7D). Equally striking was the ability of the L-type Ca^2+^ channel opener, Bay K8644, to rescue the bicuculline-evoked diminution of excitability (Figure 7C, D). Thus, Bay K8644 increased the number of cells firing spontaneous action potentials (from zero with bicuculline (n = 19) to 57% (n = 7) with Bay K8644 and bicuculline, P < 0.01, Figure 7C) hyperpolarised the membrane (from -19.5 ± 2.7mV (n = 19) with bicuculline to -43.0 ± 4.4mV (n = 13) with Bay K8644 and bicuculline (P < 0.0001, Figure 7D). 

These data suggest that ACM promotes maturation of differentiating neurons via GABA-dependent Ca^2+^ influx through voltage-gated Ca^2+^ channels. It also demonstrates the novel finding that the accelerated functional maturation of iPSCs using ACM can be replicated using a chemically defined medium of GABA and high Ca^2+^.

## Discussion

Although there have been many reports detailing protocols for generating neurons from human pluripotent stem cells, all rely on molecular and cellular markers as the primary measure of effectiveness; for example, βIII tubulin and microtubule-associated protein (MAP) 2 together with markers of neuronal subtype-specific fate determination (e.g. 5,6) Such markers are useful in determining how neurogenic each protocol might be, but they give very little indication as to whether the differentiated cells express the critical characteristics of functional neurons. Of particular importance are electrophysiological measures of V_m_ and the ability to fire action potentials, either spontaneously, or upon current injection. Using a standard, serum-free differentiation medium - containing B27 supplement, BDNF, GDNF and ascorbic acid [7] - only a small percentage of human iPSC-derived neurons were ever able to generate sAPs. However, differentiation in ACM resulted in a dramatic augmentation in the proportion of neurons which were spontaneously active, up to 74 % by week 3 (Figure 1A). Action potentials are definitive characteristics of neurons and there are several factors which are absolutely required before a cell can become spontaneously excitable. Paramount is the adequate expression of voltage-gated Na^+^ and K^+^ channels. In this study, it became clear that once particular functional expression levels of these currents had been achieved (Figure 2D), the cells could be induced to fire action potentials, and this ability was not affected by ACM (Figure 2A). Exceeding the minimum functional level of voltage-gated channels was alone not sufficient to facilitate spontaneous activity. Indeed, ACM did not evoke the observed augmentation in spontaneous activity by increasing either the extent (Figure 2B) or the magnitude (Figure 2C) of Na^+^ and K^+^ current expression *per se*. Furthermore, the activation/inactivation characteristics of the Na^+^ currents were not influenced by ACM (Figure 3B), implying that ACM did not change the ratio of different Na^+^ channel sub-types which were functionally expressed during differentiation, consistent with the demonstration that iAP thresholds were also unaffected by ACM (Figure 3B). Another requirement for the generation of spontaneous activity is that the V_m_ is sufficiently hyperpolarized to remove the voltage-dependent inactivation of Na^+^ channels. ACM evoked significant hyperpolarization of V_m_ at all weeks, most strikingly at week 3 (Figure 3A) where 74 % of ACM treated cells were spontaneously active and, across the whole cohort (298 cells), there was a strong correlation between V_m_ and the ability of cells to generate action potentials spontaneously (Figure 3A). Therefore, ACM promotes the ability of differentiating neurons to generate action potentials spontaneously by hyperpolarising the membrane to levels sufficient to remove Na^+^ channel inactivation. However, it may also be necessary that such hyperpolarized neurons must receive depolarizing stimuli, especially if they are to generate the type of activity seen *in vivo*, in order to produce the complex and rhythmic activities observed in Figure 1. Such input would be the result of synaptogenesis *in vitro*, an idea completely consistent with the observation that ACM-treated neurons were unique in their expression of spontaneous miniature synaptic currents (Figure 1D). Although miniature synaptic potentials have been shown to be increased by astrocytes [14,32] or astrocyte-secreted factors [15,33] in rodent primary neuronal cultures and long-term hES cell differentiations [1], the data herein represent the first direct observation of a short-term, contact-independent augmentation of functional maturation and synaptogenesis by astrocyte-secreted factors in neurons differentiating from human PSCs and are the first systematic determination of the electrophysiological basis of ACM-evoked enhancement of augmented spontaneous neuronal activity. 

To determine the mechanism(s) which underlie ACM-enhanced neuronal maturation, the functional expression of voltage-gated Ca^2+^ channels and GABA_A_ receptors was investigated during early stages of differentiation. Strikingly, ACM evoked Ca^2+^ channel remodelling, with large increases in both the proportion of cells expressing L-, N- and R-type channels and the magnitude of the Ca^2+^ influx through each sub-type (Figure 4). In contrast, the ontogeny of GABA_A_ receptors showed relatively little difference with ACM treatment. Thus, although the GABA_A_ responses of most cells switched from excitatory to inhibitory during differentiation (presumably via the well-established, time-dependent modulation of Cl^-^ transporters [24,28,34]), ACM changed neither the magnitude of the GABA_A_ currents nor their mode of action at any time point (Figure 5). 

Having established that ACM augmented functional expression of certain Ca^2+^ channels in the absence of significant changes in Na^+^, K^+^ and GABA_A_ channels, it seemed possible that ACM-evoked changes in functional maturation (hyperpolarized V_m_ and increased spontaneous activity) might be a result of this early Ca^2+^ channel remodeling. Indeed, inclusion of specific blockers of L-, N-, or R-type channels in the ACM resulted in depolarized V_m_ values (Figure 6B) and the complete abolition of spontaneous activity (Figure 6A), suggesting that Ca^2+^ influx is important in ACM-evoked maturation. In addition, although GABA_A_ channels are unaffected by ACM *per se* (Figure 5), but that blocking them with bicuculline also impairs maturation (Figure 6A, B), they must be providing the depolarizing stimuli for voltage-activated Ca^2+^ influx early in differentiation. This implies that ACM may augment functional maturation via enhancement of the GABA_A_-dependent, Ca^2+^ influx pathway. This was investigated directly by raising the Ca^2+^ concentration of the control differentiation medium to 1.8 mM, or by supplementing the medium with 300 μM GABA. In both cases, V_m_ values became hyperpolarised and spontaneous activity was augmented; an effect blocked by bicuculline (Figure 6C, D), nifedipine or conotoxin (Figure 7A, B). Notably, agatoxin (P/Q blockade), which is an ineffective blocker of Ca^2+^ influx (see Figure 4), was unable to affect the maturation (Figure 7A, B). Furthermore, at the cell biological level, high Ca^2+^ concentration also promoted the loss of nestin-positive cells and an increase in Tuj1 and Map2ab-positive neurons, indicative of enhanced neuronal differentation (Figure 8).

Finally, and perhaps most importantly, the ability of conotoxin (N-type blockade) to ablate the high Ca^2+^-dependent increase in functional maturation was reversed upon addition of GABA, whilst the bicuculline ablation was rescued by opening L-type Ca^2+^ channels with BayK 8644. This implies that exogenous GABA treatment negates the requirement for N-type Ca^2+^ channel-dependent endogenous GABA release [35] and that in the absence of any endogenous GABA signaling (bicuculline), direct activation of L-type Ca^2+^ channels is sufficient to enhance neuronal excitability during differentiation.

Thus, ACM enhances neuronal maturation principally by amplification of an endogenous pathway which is dependent upon GABA_A_ receptor-induced depolarization and consequent Ca^2+^ influx. This mechanism can be partially mimicked *in vitro* by modestly increasing extracellular Ca^2+^ or GABA concentrations. In the absence of a complete understanding of the factors which are secreted by astrocytes and their individual modes of action, these simple modifications can now be exploited to augment functional maturation in modified differentiation protocols. However, in contrast to ACM, this GABA-dependent mechanism is susceptible to loss of function when GABA becomes inhibitory. Therefore, in the absence of a full understanding of ACM we suggest application of Ca^2+^ channel openers offers the possibility to bypass such developmental effects and enhance maturation of PSCs as a one-step process. 
